# Development and validation of a bedside prediction score for nosocomial sepsis in the pediatric ICU: a prospective observational cohort study

**DOI:** 10.1186/cc11710

**Published:** 2012-11-14

**Authors:** LG Saptharishi, M Jayashree, S Singhi

**Affiliations:** 1 PGIMER, Chandigarh, India

## Background

Diagnosis of nosocomial sepsis (NS) is a challenge in every pediatric ICU [1,2]. There are very few studies on NS prediction in children, and those existing [3-8] have studied risk factors with emphasis on admission variables. In contrast, our study attempts to simplify the decision-making process using a dynamic scoring system based on objective criteria.

## Methods

This was a prospective study where 428 consecutive admissions, aged 1 month to 12 years, between January and October 2011 with PICU stay >48 hours, were enrolled and followed-up during their ICU stay and 72 hours thereafter. Occurrence of culture-positive nosocomial infections and relevant details were recorded. Patients with and without nosocomial sepsis were compared by chi-square test or Fisher's exact test for categorical and unpaired *t *test or Kruskal-Wallis for continuous variables. Significant predictors of NS (*P *< 0.05 on univariate analysis) were included in the binary backward stepwise logistic regression. The resultant derivation model's discrimination and calibration were assessed using the receiver operator characteristic (ROC) curve and Hosmer-Lemeshow test, respectively. The final model was transformed into a score, based on the regression coefficients. For internal validation, bootstrapping and shrinkage coefficients were used.

## Results

**Figure 1:**
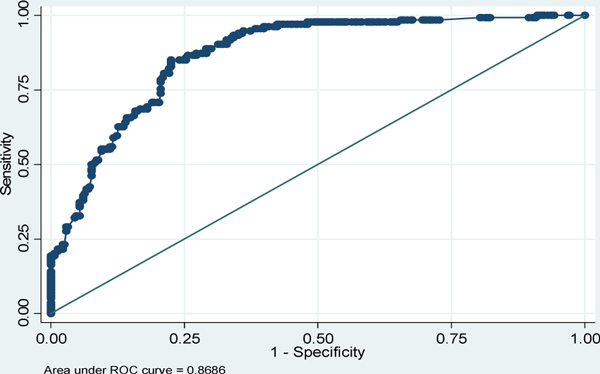
**Receiver operator characteristic (ROC) curve for the prediction model**.

Of the 428 enrolled, 17 were excluded (malignancies (14 cases), burns (one case), polytrauma (one case) and missing data (one case)). A total of 151 episodes (23.1%; 95 out of 411 children) of culture-positive NS were seen giving an incidence rate of 4.5 per 100 patient-days. Age, PRISM III score, device utilization, albumin, immunomodulator and prior antibiotic use, and intubation were significant independent predictors on multivariate analysis (Table 1). This model had an AUC-ROC of 0.87 (Figure 1). Also, the Hosmer-Lemeshow chi-square was 5.06 (*P *= 0.75) indicating good fit of the model. Based on the regression coefficients, a pediatric nosocomial sepsis prediction score (Pe-NoSeP) was developed. Probability cutoffs versus sensitivity and specificity plotting showed a cutoff of 0.38 corresponding to a score of 15. The sensitivity, specificity, positive predictive value, negative predictive value and positive likelihood ratio at this cutoff were 79.1%, 79.1%, 61.6%, 89.9% and 3.76, respectively. The accuracy of the model was 79.3% and reduced classification errors from 29.8% to 20.7%. All seven predictors retained their statistical significance after bootstrapping, confirming the validity of the score.

**Table 1:** Multivariate logistic regression analysis: independent predictors and the Pediatric Nosocomial Sepsis Prediction Score (Pe-NoSeP)

**Serial no**.	Variable	**B Coeff**.	Odds ratio (OR)	OR 95% CI	*P *value	Category	Score
1	PRISM III score	0.06	1.06	1.02 to 1.10	0.003	5 to 15	0
						16 to 26	2
						27 to 37	4
						38 to 48	7
						49 to 59	9
2	Indwelling catheter use	1.67	5.31	2.31 to 12.23	< 0.001	No	0
						Yes	6
3	Albumin transfusion	1.35	3.87	1.58 to 9.50	0.003	No	0
						Yes	5
4	Age (≤5 years/>5 years)	0.90	2.45	1.22 to 4.93	0.010	>5 years	0
						≤5 years	3
5	Immunomodulator use	1.30	3.66	1.23 to 10.94	0.020	No	0
						Yes	5
6	Intubation	1.70	5.48	2.53 to 11.88	< 0.001	No	0
						Yes	6
7	Prior antibiotic use (<4/≥4)	0.58	1.79	1.03 to 3.11	0.040	<4	0
						≥4	2

## Conclusion

The Pe-NoSeP score is a simple, easy-to-use bedside prediction model, which estimates the probability of NS in a child on a particular day and assists clinical decision-making. This tool may have diagnostic, therapeutic and preventive utilities based on its application.
